# The Value of Various Post-Processing Modalities of Diffusion Weighted Imaging in the Detection of Multiple Sclerosis

**DOI:** 10.3390/brainsci13040622

**Published:** 2023-04-06

**Authors:** Ahmad Joman Alghamdi

**Affiliations:** Radiological Sciences Department, College of Applied Medical Sciences, Taif University, Taif 21944, Saudi Arabia; a.joman@tu.edu.sa

**Keywords:** multiple sclerosis, magnetic resonance imaging, diffusion tensor imaging, diffusion kurtosis imaging, neurite orientation dispersion and density imaging, axonal diameter measurement, normal-appearing white matter

## Abstract

Diffusion tensor imaging (DTI) showed its adequacy in evaluating the normal-appearing white matter (NAWM) and lesions in the brain that are difficult to evaluate with routine clinical magnetic resonance imaging (MRI) in multiple sclerosis (MS). Recently, MRI systems have been developed with regard to software and hardware, leading to different proposed diffusion analysis methods such as diffusion tensor imaging, q-space imaging, diffusional kurtosis imaging, neurite orientation dispersion and density imaging, and axonal diameter measurement. These methods have the ability to better detect in vivo microstructural changes in the brain than DTI. These different analysis modalities could provide supplementary inputs for MS disease characterization and help in monitoring the disease’s progression as well as treatment efficacy. This paper reviews some of the recent diffusion MRI methods used for the assessment of MS in vivo.

## 1. Introduction

Multiple sclerosis (MS) is a chronic inflammatory demyelination disease that causes cognitive, sensory, and motor deterioration to the nervous system [1,2]. Injured myelin undergoes a remyelination process that may fail, and develops chronic plaques with different degrees of demyelination with or without axonal injury and gliosis [3]. The exact cause of MS is not yet known; however, it is most likely the result of either a pathogen or an aberrant response of the immune system or a combination of both [4,5]. There is also some epidemiological evidence for the environment acting in combination with genetic factors long prior to multiple sclerosis becoming evident clinically, which is also important in disease formation. MS affects more than 3 million people around the world and can affect young adults at the average age of 30 [5,6,7]. This number of MS patients is growing significantly, and thus the need for effective prevention strategies is essential [8]. MS has no cure as yet and the treatment focuses on shortening the duration of attacks as well as slowing the disease progression to minimize the symptoms [5].

Magnetic resonance imaging (MRI) is an established key imaging modality for the investigation of pathophysiology in a wide range of neurodegenerative diseases [9,10,11]. MRI not only assists in diagnosing and monitoring the disease as stated in the revised McDonald criteria [12], but also allows the evaluation of the efficacy of disease treatment [9,10,11]. Conventional MRI examinations can measure central nervous system (CNS) atrophy and MS lesions through T_1 hypo-intense, T_2 hyper-intense, and gadolinium (Gd) enhancement imaging modalities. These modalities have helped with the diagnosis, clinical management, and examination of the underlying MS disease mechanisms in clinical and pre-clinical trials [11]. However, conventional MRI has inherently low sensitivity in detecting MS gray matter (GM) and diffuse white matter (WM) damage as the imaging findings do not always agree with the degree of the disease progression [13]. This can be caused by an ongoing microstructural change in neural tissues that is invisible for conventional MRI, known as normal-appearing white matter (NAWM), which often demonstrates swelling, mild inflammation, increased expression of proteolytic enzymes, microglia activation, gliosis, and chronic injury [14]. The degree of the damage caused to NAWM differs according to the disease severity [15]. Therefore, new MRI techniques are urgently required to improve the sensitivity and specificity for measuring this type of damage.

Diffusion (also known as a Brownian motion) is a phenomenon of the thermal random movement of water molecules, which can be quantified using diffusion weighted imaging (DWI) in vivo and to ultimately define the characterization of neural activity [16,17]. In a DWI sequence, a diffusion sensitization gradient is applied on both sides of the refocusing radiofrequency pulses. A parameter called the b-value, which is proportional to the square of the amplitude and the duration of the applied diffusion gradient, determines the weighting of the diffusion images (expressed in s/mm^2^). The apparent diffusion coefficient (ADC) is a parameter that quantitatively evaluates the diffusion images and shows with a dark contrast those tissues with restricted diffusion (bright on DWI images) [18]. The free movement of water molecules that can diffuse equally in all directions is called isotropic diffusion and results in a spherical spatial displacement. To measure the isotropic diffusion coefficient, only one direction is needed [19].

**Figure 1:**
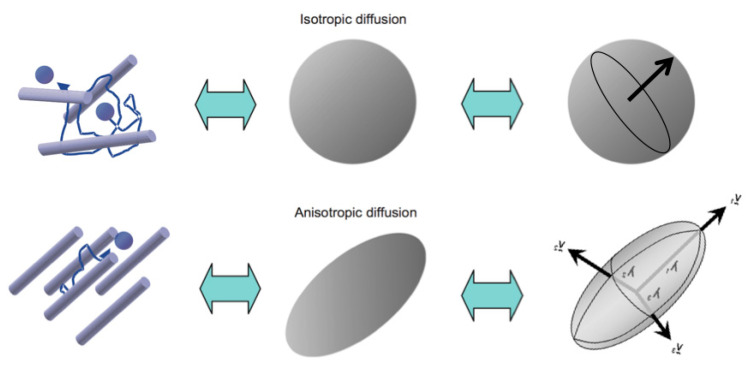
Schematic graph of isotropic and anisotropic diffusion. This figure was adapted with permission from Ref. [19]. 2023, Elsevier.

In biological tissues, the diffusion of water is complicated due to their structural boundaries [19]. Diffusion in these conditions is called anisotropic. In the central nervous system (CNS), white matter (WM) structures are highly organized, resulting in preferential water diffusion parallel to the axon direction (Figure 1) [20]. In order to obtain the anisotropic diffusion coefficient, more than one diffusion gradient direction is essential [19]. For instance, diffusion tensor imaging (DTI) requires six non-collinear directions of the diffusion-sensitized gradient measurements in addition to at least one reference measurement that is acquired with no diffusion gradient. These gradients are incorporated in a 3 × 3 symmetric matrix for each gradient direction and are called the B-matrix [21]. Thus, applying a large number of diffusion gradients will help to improve the diffusion measurement. However, this will have the drawback of increasing the scan time significantly. Moreover, the B-matrix is influenced by different factors such as the main magnetic field inhomogeneity, imaging gradients, eddy current, and other background interference [21,22]. This may lead to B-matrix spatial distribution (BSD), which should be taken into consideration and be corrected and calibrated for, as any gradient distortions of the B-matrix will cause an inaccurate diffusion measurement. Several methods have been proposed to gain a more accurate B-matrix, such as: (a) refocusing each DWI gradient before the readout gradient is turned on and refocusing each imaging gradient before the next DWI gradient [23] or (b) acquiring DWI data twice in each direction (with the given DWI gradient and with the opposite polarity [24,25]). Applying any of these methods will result in a long scanning time. The effect of imaging gradient can be decreased by using the optimal diffusion gradient scheme [26]. Moreover, most of the gradient uniformities’ effects on the B-matrix can be solved by using spherical harmonic expansion [27,28]. The BSD-DTI is an alternative calibration method of correcting the errors and setting the accurate form of the B-matrix. This can be achieved by utilizing an anisotropic phantom with well-defined structures. The phantom is rotated mechanically with a certain set of Euler angles inside the MRI scanner and then the standard DTI measurements are performed and repeated until completing the dataset. In this method, unlike previous methods, knowing all imaging sequence parameters is not required [29,30]. On the other hand, the spatial distribution can be derived in the laboratory coordinate system by a rotation transformation if the phantom has a well-known coordinate system [31]. This allows for the use of any anisotropic phantom with well-known diffusion properties with one condition of using of the phantom parameters during the whole calibration process. It is worth mentioning that experimental observations from Bammer and Markl [27] since 2003, and their following work on the BSD-DTI approach from 2008, theoretically contradict the Stejskal-Tanner (S-T) equation, where the gradient is only time-varying. Only since 2018 has the generalized S-T equation for non-uniform gradients—for which the classic S-T equation is a special case—introduced such a possibility (curvilinear space in which the gradient is constant) [32].

The accurately-produced signal contrast of DWI data can then be used as an evaluation tool with which to assess MS disease progression [18]. This review will cover some of the recent post-processing diffusion MRI techniques.

## 2. Diffusion Tensor Imaging (DTI)

**Figure 2:**
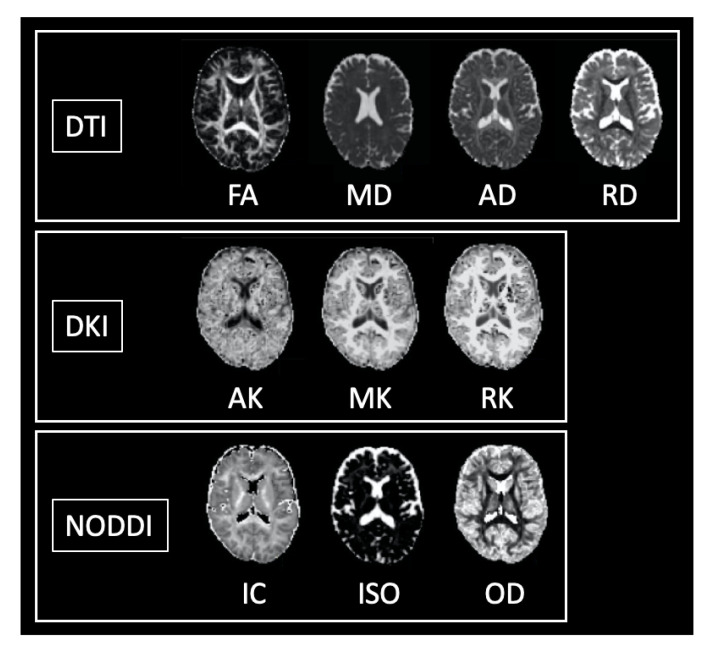
Diffusion metrics from the same participant for DTI (FA, MD, AD and RD), DKI (AK, MK and RK), and NODDI (IC, ISO and OD) [51].

DTI, since it was introduced by Basser and Mattiello [33], has proved its ability to detect abnormal changes in the brains and spinal cords of MS patients through its high sensitivity to water molecules’ motion along the tissues, and can illustrate the tissue type, integrity, architecture, and presence of barriers resulting in quantitative information about the anisotropy and orientation [34]. DTI quantitative metrics include axial diffusivity (AD), which is generally parallel to the WM fibers and measures the fast diffusion along the axons, radial diffusivity (RD), which reflects diffusion perpendicular to the fibers and provides information about diffusion between the axon myelin structures, mean diffusivity (MD), which provides a measure of the overall water motion without directionality, and finally the fractional anisotropy (FA), which is used to describe a relative diffusivity index along the largest diffusion axis of the tensor (see Figure 2) [35,36]. These parameters, in several studies, have shown changes in MS lesions as an increase in the diffusivity and a decrease in the anisotropy as a result of increased water content with a loss of myelin and axons, and the presence of gliosis [37,38,39,40,41]. Other studies have reported a consistency between a decrease in FA in lesions and a decrease in tissue coherence in adults with mild [42] and moderate MS [43], and pediatric patients with mild MS [44,45,46]. Moreover, a change in NAWM has been detected in different studies as an increase in MD and reduced FA values [43,47,48].

DTI has a drawback due to its fundamental assumption that each voxel has only one diffusion direction and accounts for the Einstein–Smoluchowski equation (λ^2^ = 2Dt), where (λ^2^) is the mean square displacement, (D) is the diffusion coefficient, and (t) is the diffusion time. This acts as the basis for the diffusion tensor image and assumes that water molecules follow a Gaussian distribution. However, water movement in neuronal tissues in vivo is inconsistent due to the presence of different compartments and barriers that cause different types of water restrictions. Thus, the theory that the water molecule is normally distributed in vivo is not correct in many cases [49,50]. This can produce some issues when analyzing relatively large voxels (>2 mm) as they could have a different tissue mixture (GM, WM, and CSF). Additionally, the presence of the cell membrane and crossing fibers may deviate the diffusion calculations from a Gaussian process [48,50]. Therefore, additional DWI modelling techniques are needed to overcome these problems. Different approaches have been developed to analyze each voxel with different components mathematically, depending on their features, to extract further knowledge about the underlying changes [18]. 

## 3. Q-Space Imaging (QSI)

QSI is an analytical model for DWI datasets utilizing high b-values. It has an advantage of not counting on the Gaussian diffusion assumption when calculating its maps compared to DTI. QSI calculates the average distance travelled of water molecules and their probability density map in one voxel during a given diffusion time with high and multiple b-values. QSI has showed a high sensitivity to NAWM changes in MS patients [52,53]. It even has a higher sensitivity than DTI in detecting pathological changes as shown in Mustafi and Harezalk’s (2019) study, where MS plaques and NAWM showed higher water molecule mean displacement values (a QSI metric that is similar to the ADC of the DTI, which illustrates the amount of water molecule restriction within the voxel) compared to DTI [54]. Moreover, myelin imaging was introduced based on QSI to monitor demyelination and remyelination processes, and helped with observing microstructural changes in MS patients in vivo [55,56,57,58]. QSI, however, is not extensively used clinically due to its need for a large number of high b-values, which results in a long scanning time [52,53].

## 4. Diffusional Kurtosis Imaging (DKI)

**Figure 3:**
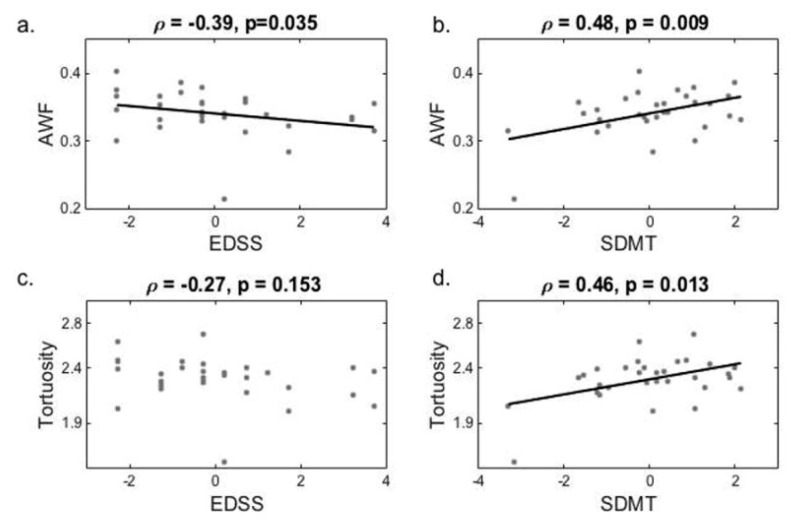
Mean axonal water fraction (AWF) and tortuosity of the corpus callosum (body) correlated with EDSS (**a**,**c**, respectively) and SDMT (**b**,**d**, respectively) scores. The best fit lines are Spearman rank for (**a**,**c**); and Pearson for (**b**,**d**) correlation coefficients. This figure was adapted with permission from Ref. [64]. 2023, Springer Nature.

DKI was introduced in 2005 by Jensen and Helpern and its metrics have been driven by QSI analysis [59]. DKI has fewer requirements for its metrics (axial kurtosis (AK), mean kurtosis (MK), and radial kurtosis (RK); see Figure 2) to be measured than QSI (it should have two b-values in addition to a b-value of zero with no fewer than 15 diffusion directions). Thus, it has the advantage of being applied clinically with a reasonable imaging time. The protocol has been utilized to collect proper DWI data for DKI analysis with an imaging time of less than 3 min [60]. One study, conducted by Bester et al., showed a decrease in the cortical mean kurtosis (one of DKI metrics) for patients with cognitive performance deficiency, where it was assessing GM rather than WM [61]. Moreover, DKI has been reported to be more accurate in detecting microstructural changes in NAWM in MS patients than DTI due to its high sensitivity measurements index [62,63]. In addition, the DKI mean axonal water fraction (AWF) parameter revealed significant correlations with the expanded disability status scale (EDSS) and the symbol digit modality test (SDMT) and the tortuosity of the extra axonal space with SDMT (see Figure 3) [64]. Another advantage of DKI is that the mean kurtosis index is not easily affected by crossing fibers in contrast to FA [62,65,66]. On the other hand, some skepticism was raised about the amount of biological tissue complexity reflected by the degree of deviation from the Gaussian distribution and the value of kurtosis in DKI due to the lack of specificity of the mean kurtosis. Thus, more studies should be performed to confirm its usefulness clinically [67].

## 5. Neurite Orientation Dispersion and Density Imaging (NODDI)

**Figure 4:**
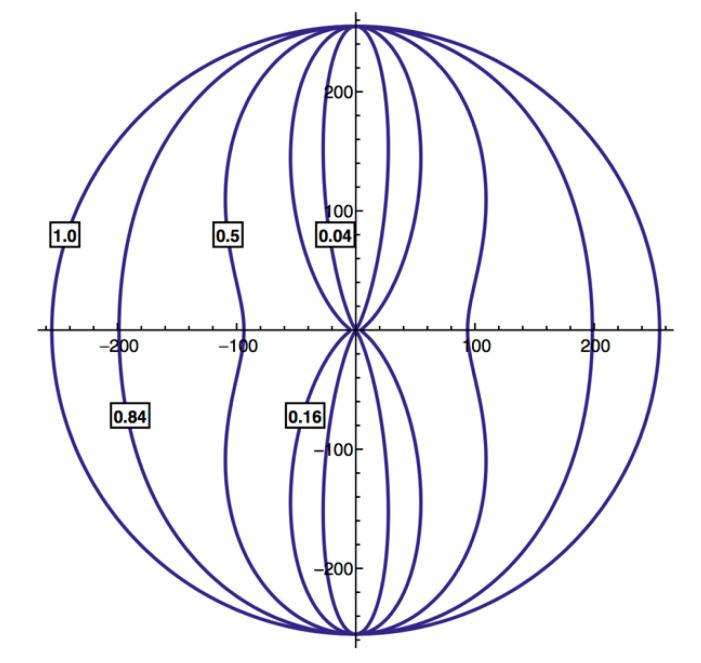
Watson distribution model. In this illustration, the mean orientation is constant but the orientation dispersion index is varied (OD ∈ [0.04, 0.16, 0.5, 0.84 and 1.0]). This model generates a symmetrical lobe, where the cross-sectional view through the axis symmetry matches its mean orientation. This figure was adapted with permission from Ref. [68]. 2023, Elsevier.

NODDI is a multi-shell, high angular resolution diffusion imaging (HARDI) technique that was developed by Zhang and Schneider [68] in 2012 for the microstructural complexity estimation of neurites in vivo. NODDI parametric indices provide more accurate markers of neurological tissues with a higher sensitivity and specificity than standard DTI and DKI indices [68,69]. This is due to DTI and DKI providing numerical representations for the information contained in DWI data without considering the underlying microstructural characteristics of biological tissue, whereas NODDI accounts for this through the orientation dispersion (OD) index to quantify neurite density [70,71]. The assumption behind the NODDI model is that water molecules in neuronal tissues follow one of three different pools: (I) a free water, which is modelled as isotropic diffusion (used to model CSF); (II) restricted water, which is modelled as sticks (used to model axons and dendrites); and (III) hindered water, which is modelled by anisotropic diffusion Gaussian distribution (used to model extracellular space, neuronal cell bodies, and glial cells) [51,72]. This will result in the formation of three indices for the NODDI model including isotropic diffusion fraction (ISO), orientation dispersion fraction (OD), and intracellular fraction (IC) (see Figure 2) [68]. The Watson distribution model (Figure 4) is used to calculate the orientation of the sticks to calculate the dispersion, which ranges from highly parallel axons (e.g., the corpus callosum) to widely dispersed dendrites (e.g., GM) [50,68]. The required DWI data for NODDI analysis are similar to the DKI requirement. Many studies have used NODDI to evaluate brain MS lesions and NAWM [73,74,75,76,77,78,79,80]. These studies had common findings in IC reduction, which suggests neurite degeneration and reduced density, in MS plaques. Moreover, NAWM showed a decrease in the IC compartment but was not similar to demyelinated regions. A post-mortem study of the human brain showed a good agreement between IC map with histology in the hippocampus axonal pathways (perforant path, Schaffer collaterals, and mossy fibers) [81]. Another post-mortem study was performed on the human spinal cord for MS patients and showed a reduction in the OD map with an excellent agreement with histology findings [82].

NODDI provides sufficient information to measure neurite morphology in vivo, as well as enabling potential clinical application among many proposed models [68,83]. However, there is some skepticism with regard to using NODDI in clinical research as its analysis may not always fit correctly with the underlying tissue pathological conditions [83]. Thus, further studies on more clinical cases and comparing them with histopathology, EDSS, and disease prognosis are essential for confirmation [78].

## 6. AxCaliber

Neuronal signal conduction has been found to be directly correlated with axon diameter. Hence, axon diameter controls the transmission capacity within the central nervous system (CNS) and peripheral nervous system. Axons with a large diameter are found in bundles that require a fast response, such as motor nerves, while axons associated with pain and temperature controls have a smaller neuronal path and have a slower response [84].

Whenever axons are damaged as a result of injury or autoimmune or neurodegenerative diseases (such as MS), the patient’s quality of life will be dramatically affected, resulting in sensory and/or motor deficits [85]. MS pathology affects smaller axons first [86]. Therefore, measuring the axon diameter distribution is important for detecting the effect of the disease on neuronal function. Until recently, such information was only accessible through histological examination using invasive tissue biopsy. However, a novel method called AxCaliber was developed by Assaf and Blumenfeld-Katzir [84] in 2008 that enabled the non-invasive measurement of axon diameter distribution in vivo using diffusion MRI. The idea behind it was that diffusion restriction will be experienced differently by axons with different diameters at different diffusion times. Thus, it is possible to estimate the axon diameter distribution by modelling the multi-diffusion time data as a gamma function [87]. AxCaliber is considered an extended framework of the composite hindered and restricted model of diffusion (CHARMED) approach, which was developed by Assaf and Basser [88] in 2005 [84]. It uses a series of DWI-stimulated echo images to extract the axonal diameter distribution and volume fraction for each voxel [85,89]. The first launched AxCaliber model was achieved by applying diffusion gradients perpendicular to the axons’ direction with different diffusion times [87]. This approach made its use limited to highly paralleled WM tracts such as in the corpus callosum of the brain [87,90,91,92] and the WM tracts in the spinal cord [85,93]. Another imaging model (called ActiveAx) was published in 2010 that studies the axonal features with few requirements for the acquisition protocol. The advantage of ActiveAx is that it is not affected by the axon’s orientation. However, unlike AxCaliber, ActiveAx estimates only the mean axon diameter rather than the full axon diameter distribution [87]. Later, in 2019, De Santis and Herranz [94] developed AxCaliber to extract axonal diameter measurements across the whole brain and validated the axonal diameter calculated through AxCaliber with histological findings and reported a linear fitting with a value of R^2^ = 0.99.

Axons with small diameters are at risk of injury when demyelination occurs. This results in a larger apparent axon diameter calculation for NAWM and lesion areas, which has been found to have a strong correlation with EDSS performance [92]. Several studies have reported axonal diameter increase in lesions and NAWM [95] as well as a decrease in axonal density [92,96] of the corpus callosum in MS patients compared to healthy controls. Additionally, an increase in axon diameter with a reduction in water molecule restriction was reported for the whole brain using AxCaliber in MS compared to healthy controls [94].

AxCaliber has been argued to be overestimating the axon diameter [78]. Gradient strength is essential to solving this issue as it requires a high gradient coil (e.g., 300 mT/m that has been used in studies by Duval and McNab [85], Huang and Fan [92], De Santis and Herranz [94], and Huang and Tobyne [95]), whereas the clinical MRI scanners have a range of 30–80 mT/m [78,85].

## 7. Future Perspective

A more detailed assessment of MS pathology, such as disease severity and treatment efficacy, can be extracted by combining different advanced diffusion models. The main challenge facing advanced diffusion MRI models is the amount of required data, and sometimes specific hardware, for the analysis to occur, leading to a long scanning time when compared to DTI. One of the solutions is to reduce the diffusion time by replacing the long diffusion-sensitizing gradients used in pulsed gradient spin-echo sequences with rapidly oscillating gradients [97,98]. Another solution is using an imaging protocol that serves different diffusion analysis methods, such as hybrid diffusion imaging (HYDI) [99]. The HYDI scheme consists of multiple shells of DWI. These shells can be used for characterizing the signal behavior with low, moderate, and high diffusion weighting, and could be facilitated to be analyzed using different analysis strategies such as DTI, Q-Space, DKI, and NODDI, resulting in comprehensive findings for the same DWI data.

The recent developments in manufacturing and designing gradient coils have helped to achieve higher b-values as low gradient systems prevent the achievement of a high b-value. This also led to the achievement of a constant gradient field, which allowed for the acquisition of an adequate stimulated echo from very short diffusion times, as well as the application of very short diffusion encoding times [100]. For an example, a maximum b-value with a gradient system of 0.5 T/m can be achieved with a diffusion time of 50 ms; using a stronger gradient system by a factor of 10 (with a capability of 5 T/m), the diffusion time can be shortened by a factor of 20 (decreasing the diffusion time to 2.5 ms) [101]. Moreover, the increase in the resolution of DWI data has led to deeper insights into how functional and structural properties of the brain are coupled through combining DWI with electro-encephalography (EEG) [102].

In very recent years, the number of new diffusion approaches has increased. Nuclear magnetic resonance (NMR) diffusometry is considered a new, unique promising technique for evaluating nanomedicine, whereas nanoparticle-based vaccines used against COVID-19 throughout the pandemic have accelerated the development of nanomedicines in the coming years—NMR diffusometry will be a necessary tool with which to investigate their fate in bioenvironments. NMR diffusometry could complete a range of assays for different drug assessment methods and overcome some of their drawbacks. This application is still in its early development stage but should make a better contribution to understanding the characterization and bioenvironment interaction complexity of nanomedicine in biological systems. NMR diffusometry may allow for new MS treatment approaches as, when nanomedicine is administered, the administration route until it reaches its biological target illustrates the mechanisms of interactions between the drug and the tracer (either encapsulated, entrapped, dissolved, covalently bonded, or adsorbed on the surface) or the drug release process (inclusion and location), and the therapeutic results of where and when the tracer will deliver the drug [101]. Another recent imaging technique is diffusion basis spectrum imaging (DBSI). This technique combines the spectrum of isotropic diffusion tensors (composed of a restricted fraction, which reflects cellularity, and a nonrestricted fraction, which reflects the extra-axonal environment correlated with tissue loss, inflammation, and cerebrospinal fluid partial volume) with discrete anisotropic diffusion tensors (composed of axial diffusivity (which represents residual axon integrity), radial diffusivity (which represents myelin integrity of the remaining axons), and fiber fraction (which represents apparent axonal density)) to investigate MS subtypes’ neuropathology. DBSI has provided preliminary evidence that supports its ability to become a potential non-invasive biomarker of remyelination and a neurodegenerative measurement tool for MS pathology [103]. Another technique showed that DWI can assess WM connections, revealing topological alterations in MS using structural network-based approaches [104,105] and subnetwork-based approaches [106]. These network analyses could serve as potential biomarkers that reflect the state of the brain network more accurately through diagnosing and monitoring disease progression and assessing MS treatment effects. Finally, it is important for a certain consensus on specific DWI parameters (such as repetition time, echo time, number of averages, small and large deltas, number of b-values, b-matrix size, image resolution … etc. for all other required DWI parameters for a certain magnetic field strength) to be created and followed to minimize and overcome some of the issues that may arise from using inadequate parameters leading to inaccurate diffusion measurement.

## 8. Conclusions

This article has surveyed the potential of DWI in MS pathology. Several valuable modalities were presented to highlight their contributions to better understanding the outcomes of different DWI data models. This can help with diagnosis and improving disease management in MS patients through empowering clinical trials as well as monitoring treatment efficacy. Many other interesting modalities would certainly deserve to be mentioned in this review; however, the aim was not to be detailed but rather illustrative of the potential of DWI.

## Data Availability

This review has no data to be shared.
